# The Effects of Postharvest Treatments and Sunlight Exposure on the Reproductive Capability and Viability of *Phyllosticta citricarpa* in Citrus Black Spot Fruit Lesions

**DOI:** 10.3390/plants9121813

**Published:** 2020-12-21

**Authors:** Providence Moyo, Paul H. Fourie, Siyethemba L. Masikane, Régis de Oliveira Fialho, Lindokuhle C. Mamba, Wilma du Plooy, Vaughan Hattingh

**Affiliations:** 1 Citrus Research International, P.O. Box 28, Nelspruit 1200, South Africa; phfourie@sun.ac.za (P.H.F.); mgcogcoma@gmail.com (S.L.M.); lindo@cri.co.za (L.C.M.); wilma@cri.co.za (W.d.P.); vh@cri.co.za (V.H.); 2 Department of Plant Pathology, Stellenbosch University, Private Bag X1, Stellenbosch 7602, South Africa; 3 Department of Plant Pathology and Nematology, University of São Paulo, Piracicaba 13418-900, SP, Brazil; rfialho@usp.br; 4 Department of Horticultural Science, University of Stellenbosch, Private Bag X1, Stellenbosch 7602, South Africa

**Keywords:** CBS, pycnidiospores, dispersal, control, fungicide, wax, pathway

## Abstract

Citrus black spot (CBS) is caused by *Phyllosticta citricarpa*, which is classified as a quarantine organism in certain countries whose concerns are that CBS-infected fruit may be a pathway for introduction of the pathogen. This study evaluated the reproductive capability and viability of *P*. *citricarpa* under simulated conditions in which the whole fruit, peel segments, or citrus pulp with CBS lesions were discarded. Naturally infected ‘Midknight’ Valencia orange and ‘Eureka’ lemon fruit, either treated using standard postharvest sanitation, fungicide, and wax coating treatments or untreated, were placed into cold storage for 5 weeks (oranges at 4 °C and lemons at 7 °C). Thereafter, treated and untreated fruit were incubated for a further 2 weeks at conditions conducive for CBS symptom expression and formation of pycnidia. The ability of pycnidia to secrete viable pycnidiospores after whole fruit and peel segments or peel pieces from citrus pulp were exposed to sunlight at warm temperatures (±28 °C) and ±75% relative humidity levels was then investigated. The combination of postharvest treatments and cold storage effectively controlled CBS latent infections (>83.6% control) and pycnidium formation (<1.4% of lesions formed pycnidia), and the wax coating completely inhibited pycnidiospore release in fruit and peel segments. Pycnidiospores were secreted only from lesions on untreated fruit and peel segments and at low levels (4.3–8.6%) from peel pieces from pulped treated fruit. However, spore release rapidly declined when exposed to sunlight conditions (1.4% and 0% after 2 and 3 days, respectively). The generally poor reproductive ability and viability of CBS fruit lesions on harvested fruit, particularly when exposed to sunlight conditions, supports the conclusion that citrus fruit without leaves is not an epidemiologically significant pathway for the entry, establishment, and spread of *P. citricarpa*.

## 1. Introduction

Large quantities of citrus fruit are exported every year from citrus black spot (CBS) endemic countries into the European Union (EU), which has listed *Phyllosticta citricarpa* (the causal organism of CBS) as a quarantine pathogen. Despite previous expert reports to the contrary [1,2], the EU remains concerned that pycnidiospores produced in pycnidia occurring in CBS lesions on citrus fruit, peel, and citrus byproducts may persist as a viable source of *P. citricarpa* inoculum. The EU then concluded that infected fruit is a moderately likely pathway for introduction of the pathogen [3].

CBS causes superficial and cosmetic lesions on mature citrus fruit and can also lead to premature fruit drop under high-disease conducive conditions when the disease is poorly controlled [4]. Infection in the field occurs primarily through ascospores released from pseudothecia produced and matured on dead infected leaves on the orchard floor [5,6,7]. Pycnidiospores ooze from mature pycnidia in a gelatinous mass during wet conditions and are dispersed by water for short distances (<1 m) downward from the source to cause new infections [8,9,10,11]. Because of their dispersal limitations, pycnidiospores are considered to play a relatively minor role in the epidemiology of CBS compared with the airborne ascospores [8,12]. Infection from pycnidiospores produced in pycnidia in CBS lesions within the tree plays an important role in the epidemic, especially under high-rainfall conditions [9,10,13,14,15,16] and orchard management practices that contribute to inoculum buildup in the tree (e.g., overlapping fruit set and poor pruning) [5,9].

Infection of susceptible tissues is followed by a long period of latency before symptoms are observed [5,17]. Various types of CBS lesions are expressed on the rind of maturing fruit, including hard spot, freckle spot, false melanose or speckled blotch, virulent spots, and lacey and tan spot. Pycnidia are, however, only formed in hard spot [8,11,18,19,20,21,22], freckle spot [19,23] and virulent spot lesions [8,19,22]. Lesions on fruit only contain pycnidia and never pseudothecia [5,17].

The ability of *P. citricarpa* pycnidia to secrete pycnidiospores after reddish hard spot lesions were exposed to sunlight at mild to warm temperatures was investigated on grapefruit and Valencia orange peel segments [24]. Pycnidiospore secretion was observed to cease completely after sunlight exposure of grapefruit and Valencia orange peel segments for 4 h and 6 h, respectively [24]. The fruit used in this study were, however, not subjected to packhouse treatments and subsequent cold storage, as is common shipping protocol for export fruit.

Postharvest lesion development required long incubation periods at high humidity, with small red and sunken lesions observed after 5–7 days and pycnidium formation after 9–13 days [25]. The reproductive capability of *P. citricarpa* on fruit lesions and the length of time that mycelium remains viable in lesions diminished over time, particularly in hard spots with dark brown or black margins. Postharvest treatments followed by cold storage significantly reduced the viability of lesions and rendered resultant pycnidiospores dead [25,26]. Schreuder et al. (2018) [27] also reported moderate to high levels of control of latent *P. citricarpa* fruit infections by the combination of standard packhouse fungicide treatments (including pre-packhouse drench, packhouse dip, and brush application of a wax coating). Cold storage subsequent to packhouse treatments further improved the levels of control. Of the new lesions, an average of 10% on lemons and 15% on oranges formed pycnidia, indicating that *P. citricarpa* generally had a low reproductive capability in fruit lesions, which was, in most cases, further diminished by the combination of treatments followed by cold storage [27]. The latter was determined from the presence of pycnidia only, and it was not determined whether the pycnidia were capable of yielding pycnidiospores or whether the spores were alive or dead.

The purpose of this study was, therefore, to evaluate the viability and reproductive capability of lesions of *P. citricarpa* on fruit, including whole fruit, peel segments, and peel pieces in pulped fruit subjected to postharvest fungicide treatments and cold storage and later exposed to sunlight, and more specifically, to study the ability of pycnidia to yield pycnidiospores.

## 2. Results

### 2.1. Effects of Treatments on New CBS Lesion Development

**Figure 1:**
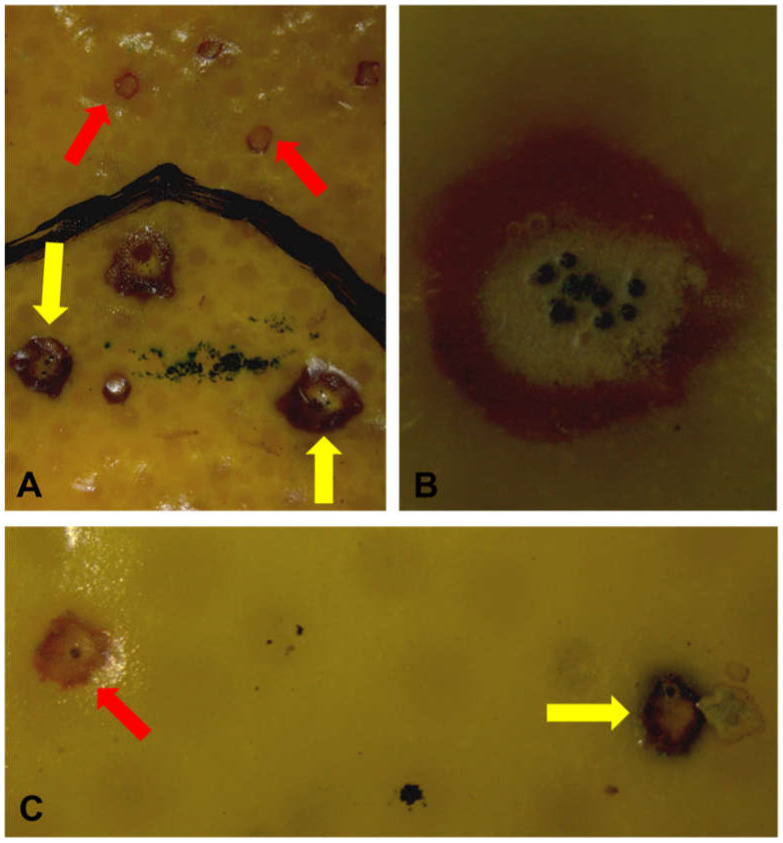
Citrus black spot (CBS) lesions on fruit. (**A**) Newly developed and old CBS lesions on a ‘Midknight’ Valencia orange fruit subjected to a complete packhouse treatment regime consisting of pre-packhouse drench, chlorine wash, fungicide dips and wax application. (**B**) New CBS lesion with pycnidia on untreated ‘Eureka’ lemon fruit. (**C**) New and old CBS lesions on untreated ‘Eureka’ lemon fruit. New and old CBS lesions are shown by red and yellow arrows, respectively.

New CBS lesions were observed on both treated and untreated fruit after incubation at optimum conditions for symptom expression. Treatments consisted of standard packhouse actions (pre-packhouse drench, chlorine wash, fungicide dips, and wax application) before exposure to a 5-week period of cold storage. Untreated fruit were subjected to cold storage without packhouse treatments. Both hard spots and red spots developed during incubation, but the latter were more abundant (Figure 1).

Analysis of variance (ANOVA) of data from lemon trials indicated a significant treatment × season interaction (*p* < 0.0001) for number of new lesions and a significant treatment effect (*p* < 0.0001) for percentage of new lesions with pycnidia. The combination of postharvest treatments and cold storage significantly (*p* < 0.0001) reduced the numbers of new CBS lesions in both seasons (1.30–2.10), with untreated fruit in the 2019 season having significantly more new lesions (17.60) than fruit in 2020 (7.90) (Table 1). The mean percentage of new lesions with pycnidia was significantly higher on untreated compared to treated lemon fruit (6.61% vs. 1.40%) (Table 1).

ANOVA of data from oranges indicated significant treatment effects (*p* < 0.0001) observed for a number of new lesions and a percentage of new lesions with pycnidia. Similar to observations in the lemon trials, a significantly higher mean number of new CBS lesions was observed on untreated oranges (14.21) compared to oranges treated with postharvest treatments and cold storage (2.33). The mean percentage of new lesions with pycnidia was significantly higher on untreated (4.43%) compared to treated (0.77%) oranges (Table 1).

### 2.2. Pycnidiospore Release and Viability after Sun Exposure of CBS Lesions

#### 2.2.1. Whole Fruit and Peel Segments

ANOVA of spore release data from lemon trials indicated a significant treatment × sun-hours interaction (*p* = 0.044) for spore release. Spore release from whole fruit and peel segments did not differ significantly (*p* = 0.085). Packhouse treatments and cold storage completely inhibited pycnidiospore release as none of the pycnidia released spores. Microscopic observations revealed that pycnidia on treated fruit were covered with the wax coating. Spores were released from 100% of the inspected pycnidia on untreated whole fruit and peel segments that were not exposed to sun (0 hours). Sun exposure led to a significant reduction in the proportion of pycnidia-releasing spores (82.80%, 81.00%, and 74.10%) after 2 h, 4 h, and 6 hours of sun exposure (Table 2).

ANOVA of spore release data from orange trials also indicated a significant treatment × sun-hours interaction (*p* = 0.027) for spore release and no significant effect of whole fruit and peel segments (*p* = 0.293). Similarly to lemons, none of the inspected pycnidia on treated fruit or peel segments released any spores. Spore release on untreated fruit significantly declined from 84–82% between 0 and 2 h of sun exposure to 59% and 58% after 4 and 6 h of sun exposure, respectively.

In the preliminary lemon trial in 2019, germination of pycnidiospores was not observed within 48 h of plating and water agar plates were discarded. Therefore, germination was not successfully evaluated. In 2020, the water agar plates were incubated longer and pycnidiospore germination on water agar was recorded from the fourth day after plating. Due to contamination with various fast-growing fungi on most of the oatmeal agar plates in which single germinating spores were plated, results from oatmeal agar plates could not be obtained. Subsequently, the viability of pycnidiospores was only determined by recording germination of pycnidiospores as observed on water agar plates.

ANOVA of percentage spore viability data from lemons (untreated fruit/peel and 2020 only) indicated significant whole fruit/peel and sun-exposure effects (*p* = 0.013 and < 0.0001, respectively). A significantly higher spore viability percentage was recorded on whole fruit (43.24%) compared with peel segments (26.67%). Spore viability was significantly lower after exposure to sunlight (26.67–18.75%) compared with 65.0% on fruit or peel not exposed to sunlight (results not shown). ANOVA of percentage spore viability data from oranges (untreated fruit/peel only) indicated a significant sun-exposure effect (*p* = 0.013) and no difference between whole fruit and peel (20.0% and 16.5%, respectively; *p* = 0.532). Spore viability was 23.81% to 20.34% at 0 to 4 h, whereafter it declined significantly to 3.45% after 6 h of sun exposure (results not shown).

#### 2.2.2. Citrus Pulp

ANOVA of the percentage of lesions with pycnidia data from lemon pulp trials indicated a significant exposure time effect (*p* < 0.0001), and no significant treatment effect (*p* = 0.249). Significantly fewer pycnidia were recorded after exposure for 1 to 3 days (37.93–36.29%) compared with lesions inspected on the unexposed pulp (44.36%). ANOVA of the percentages of pycnidia from these lesions that released pycnidiospores indicated a significant treatment × exposure time interaction (*p* < 0.0001). The 54.3% spore release proportion recorded from pycnidia on untreated pulp peel segments that were not exposed to the sun significantly declined to 21.4% after 1 day of exposure and to 8.6% and 3.6% after 2 and 3 days, respectively. Spore release from pycnidia on pulp peel segments from treated fruit declined from 4.3% on unexposed fruit to 0% after 3 days of exposure (Table 3). No significant effects (*p* > 0.124) were observed for spore viability, which ranged between 0% and 20.0% on treated pulp segments and 20.0% and 58.3% on untreated pulp segments (results not shown).

ANOVA of percentage lesions with pycnidia data from orange pulp trials indicated a significant treatment effect (*p* = 0.001) and no significant exposure time effect (*p* = 0.145). Significantly fewer pycnidia were recorded on pulp peel pieces from treated fruit (44.4%) compared to untreated fruit (48.9%). ANOVA of percentages pycnidia from these lesions that released pycnidiospores indicated a significant treatment × exposure time interaction (*p* = 0.014). Significantly fewer pycnidia released spores on unexposed peel pieces from treated fruit (8.6%) than untreated fruit (19.3%). These percentages were similar after 1 day of exposure (7.9% and 17.9%, respectively), whereafter it declined, respectively, to 1.4% and 2.9% after 2 days of exposure and 0% after 3 days (Table 3). No significant effects (*p* > 0.299) were observed for spore viability, which ranged between 16.7% and 50.0% (results not shown).

### 2.3. Verification of CBS Lesion Diagnosis

All the 64 lesions that were tested using quantitative polymerase chain reaction (qPCR) to verify the accuracy of the visual diagnosis of CBS lesions were confirmed to be caused by *P. citricarpa*. All of the DNA extracts from the representative CBS lesions on lemons and oranges were positive for *P. citricarpa*, with Cq values less than 30. The high quality and consistency of DNA extracts from these CBS samples was shown by the COX Cq values ranging between 15 and 25 (results not shown).

## 3. Discussion

The generally poor reproductive capability and viability of *P. citricarpa* in harvested citrus fruit lesions was clearly demonstrated in this study under simulated conditions if the whole fruit, peel segments, and citrus pulp were discarded and left under natural sunlight conditions. The reproductive capability of lesions in fruit and peel segments was completely diminished by standard packhouse treatments and cold storage, while peel segments in citrus pulp from treated fruit had a very low reproductive capability, which diminished completely after 3 days of exposure to sunlight.

Standard postharvest treatments are aimed at maintaining fruit quality and reducing postharvest decay [28,29] and include dip treatments in sanitizers and fungicides, wax and fungicide coating applications, and cold storage. Our study confirmed previous reports of moderate to high levels of CBS control by these treatments [26,27,30,31]. The control of latent CBS infections ranged from 83.6% on oranges to 88.1% on lemons. The poor reproductive capability of fruit lesions reported by these studies was also confirmed: Only 4.4% and 6.6% of lesions formed pycnidia on untreated orange and lemon fruit, respectively, which declined to 0.8% and 1.4% on treated fruit. These levels were similar to the 10–15% and <0.8% lesions producing pycnidia on untreated and treated fruit reported by Schreuder et al. (2018) [27].

Pycnidiospore release from pycnidia in CBS lesions were effectively stimulated using a droplet of Valencia juice and citric acid solution [24,27]. Spore release was recorded from >84% of pycnidia on untreated fruit or peel segments. This proportion significantly declined after sunlight exposure, but not to the zero levels reported by Schutte et al. (2014) [24]. Viability of the released spores declined 3.5- and 7-fold from 65% and 24%, respectively, on lemon and orange fruit and peel segments after sunlight exposure. Spore viability was significantly poorer on lemon peel segments than on whole fruit. The slightly higher incidence of spore release and viability on whole fruit could be due to the higher moisture content on whole fruit compared to excised peel segments which dried out rapidly.

Pycnidiospore release from pycnidia in CBS lesions on packhouse treated fruit or peel segments was completely prevented. The microscopic observations in our study confirmed observations by Schreuder et al. (2018) [27] that the wax coating applied on treated fruit physically prevented the release of pycnidiospores. Wax coating application of citrus fruit is a standard practice for fruit destined for export markets to prevent moisture loss, maintain fruit quality, and protect against postharvest decay through the inclusion of postharvest fungicides [29]. Our study did not evaluate the effects of wax coatings alone and cannot conclude that the physical coating effect alone prevented spore release. Schreuder et al. (2018) [27] showed that wax only and wax and fungicide resulted in moderate levels of CBS control (37–62%) and postulated that the wax coating effect to maintain healthy fruit physiology might enable host response [21] to control latent infections.

Citrus tissue type (whole fruit or peel segments) did not significantly influence the incidence of spore release. The effects of packhouse treatments and sunlight exposure on spore release from pycnidia on peel pieces in citrus pulp were studied in separate trials and could not statistically be compared with whole fruit and peel segments. Whereas >84% of pycnidia released spores in untreated whole fruit and peel segments, on average, only 54.3% and 19.3% were released from peel pieces of pulped lemons and oranges that did not receive any packhouse treatments or sunlight exposure. These proportions respectively declined to 3.6% and 0% after 3 days of sunlight exposure. In our trials on treated whole fruit and peel segments, zero spore release was observed from pycnidia. From the pulp peel pieces from treated fruit that were not exposed to sunlight, low levels of spore release were observed (4.3% and 8.6%), but these levels declined to 1.4% and 0% after 2 and 3 days of sunlight exposure, respectively. Low levels of spore release from treated peel pieces might possibly be attributed to cracks or degradation of the wax layer by the pulping process and should be investigated further. Spore viability on unexposed treated and untreated lemon and orange fruit peel pieces was similar but declined to 0% on treated lemon fruit after 2 days of sunlight exposure. This decline was not evident on oranges.

Our study clearly demonstrated the rapid decline in viability and reproductive ability of *P. citricarpa* in CBS lesions on whole fruit, peel segments, and peel pieces in citrus pulp when exposed to sunlight conditions. Warm temperatures (±28 °C) and fairly moist humidity levels (±75% RH) were recorded during these exposure periods. These conditions were more humid than the low-humidity conditions (<50% RH) required to dry citrus waste in open-air drying facilities [3,32,33]. Survival and reproduction of *P. citricarpa* under such conditions can be expected to be markedly poorer, if not nonexistent, considering that high-humidity conditions are required for lesion and pycnidium formation [8,34]. Wang and Dewdney (2015) [34] did not observe any pycnidium formation following incubation below 72% RH, and Kiely (1948) [8] observed spermagonia development above 73% RH only. Conversely, longer survival and reproductive periods might be conceivable under milder and more humid conditions and need to be investigated.

Standard postharvest treatments, as commonly employed for fresh citrus fruit (a combination of postharvest sanitation and fungicide treatments, including wax application and cold storage), effectively controlled CBS latent infections and pycnidium formation, and the wax coating completely inhibited pycnidiospore release in fruit and peel segments. This clearly indicates that infected packhouse treated fruit is not a viable pathway for *P. citricarpa* spread via the consumer pathway. In this study, the poor reproductive capability of CBS fruit lesions was clearly demonstrated, and *P. citricarpa* viability rapidly declined after sunlight exposure of whole fruit, peel segments, and citrus pulp. Low levels of viable pycnidiospore release were observed from pycnidia in lesions on peel pieces in citrus pulp from treated fruit (<8.6%), and spore release and viability rapidly declined when exposed to sunlight conditions. Infected peel pieces in citrus pulp cannot, however, be considered as an epidemiologically probable pathway for *P. citricarpa* spread, since the high temperatures and low humidity commonly employed to dry citrus pulp [3,32,33] are not conducive to pycnidium formation and spore release [8,11,34]. Moreover, pycnidiospores are effectively water-dispersed for short and downward distances (<1 m) from the source [8,9,10,11] and effective dispersal from a discarded citrus pulp to a susceptible host is highly unlikely. Our study therefore supports the conclusion that citrus fruit without leaves do not represent an epidemiologically significant pathway for the entry, establishment, and spread of *P. citricarpa* [1,2].

## 4. Materials and Methods

### 4.1. Fruit Harvesting, Treatments, and Incubation

‘Eureka’ lemons and ‘Midknight’ Valencia oranges were used in the trials because of their high susceptibility to *P. citricarpa* infection [8]. Fruit with CBS lesions were collected from orchards located in Nelspruit, Mpumalanga Province, South Africa. A complete packhouse treatment regime outlined by Schreuder et al. (2018) [27], consisting of pre-packhouse drench, chlorine wash, fungicide dips, and wax application was applied on fruit before all the CBS lesions on both fruit types were marked with a permanent marker. The drench mixture contained the following fungicides in the order in which they were added into 120 L of tap water: Thiabendazole (1000 mg L^−1^, ICA International Chemicals, Stellenbosch, South Africa), pyrimethanil (1000 mg L^−1^, ICA International Chemicals, Stellenbosch, South Africa), propiconazole (600 mg L^−1^, ICA International Chemicals, Stellenbosch, South Africa), and 2,4-dichlorophenoxy acetic acid (250 mg L^−1^, Citrashine, Johannesburg, South Africa). After drenching, fruit were kept for 24 h to dry before being subjected to a fungicide dip consisting of 500 mg L^−1^ imazalil sulphate (ICA, International Chemicals, Stellenbosch, South Africa). Thereafter, a polyethylene-based wax (18% solids; PolyOrange, Decco Citrashine (Pty) Ltd., Johannesburg, South Africa) mixed with imazalil (2000 mg L^−1^) was applied on fruit.

After treatment and marking of lesions, fruits were placed into cold storage for 5 weeks. ‘Midknight’ Valencia fruit were stored at 4 °C, whereas ‘Eureka’ lemons were stored at 7 °C. Untreated fruit were subjected to cold storage without packhouse treatments. Preliminary trials were conducted on lemons between March and May 2019 and further trials were conducted from August to October 2019 and March to May 2020 for oranges and lemons, respectively.

After 5 weeks in cold storage, all fruit were incubated for a further 2 weeks at conditions conducive for expression of symptoms and formation of pycnidia (25–27 °C under constant light and high (>80%) humidity) [25,35,36]. The new CBS lesions expressed within the period of incubation were counted and the number of new lesions that developed pycnidia was also recorded. Due to rot caused by postharvest pathogens, such as *Penicillium* spp. during the incubation period, the initial number of lemon and orange fruit used in the trials was reduced by the time evaluation for new CBS lesion development was carried out. Of the lemons, 1080 untreated and 2171 treated fruits were evaluated for new CBS lesion development in 2019. In the 2020 trials, 1068 untreated and 2268 treated lemon fruits were evaluated while 1452 untreated and 2952 treated orange fruits were evaluated for new CBS lesions.

Fruit with new lesions containing pycnidia were selected for use in further experiments to determine the ability of pycnidia to yield pycnidiospores after exposure to direct sunlight and the viability of secreted spores. In these further trials, 72 fruit with lesions containing pycnidia were used for both untreated and treated lemons in 2019, and 160 untreated and 48 treated lemons were used in 2020. With the orange trials, 400 untreated and 120 treated orange fruits were used.

### 4.2. Sunlight Exposure of Whole Fruit and Peel Segments

Both treated and untreated fruit with new lesions containing pycnidia were divided into two groups: The edible parts of 1 group were removed such that only the fruit peel segments were used in the subsequent trials while fruit were kept whole for the other group. Whole fruit and fruit peel segments (approximately 3.5 cm × 2.5 cm) with new lesions containing pycnidia were exposed to direct sunlight (flavedo facing the sun). One set of whole fruit and peel segments were subjected to immediate examination for pycnidiospore release without exposure to the sun (0 h), while the other sets of fruit and peel segments were examined for pycnidiospore release after 2 h, 4 h, and 6 h of exposure to natural conditions in direct sunlight. Temperature and relative humidity were measured using data loggers (Tinytag, Gemini Data Loggers Ltd., Chichester, United Kingdom). Temperatures ranged from 21.1°C to 38.7 °C (average = 28.4 °C) and relative humidity ranged between 44.2% and 100% (average = 75.1%). The data loggers used during the trials in Nelspruit (South Africa) only measured temperature and humidity. Other factors, such as solar radiation and evapotranspiration, could not be measured onsite. Therefore, solar radiation and evapotranspiration data from an iLeaf weather station (Hortec, Cape Town, South Africa), located approximately 18 km away from the experimental location, was used. This weather station has the same elevation above the sea level (677 m) and the same north-facing aspect. The values for solar radiation and evapotranspiration ranged between 0.80 MJ/m^2^ and 3.13 MJ/m^2^ (average = 2.17 MJ/m^2^) and 0.14 mm and 0.67 mm (average = 0.46 mm), respectively. No rain was recorded during the trials. For each sun-exposure time period investigated, 1 lesion per whole fruit or peel segment was examined for pycnidiospore release. Trials were conducted on lemons and oranges in 2019 and 2020. Fruit numbers used per treatment varied depending on the availability of fruit with CBS lesions. In 2019, the lemon trial consisted of 3 replicates. For each replicate, 3 whole fruit and 3 peel segments were examined per sun-exposure time period for both untreated and treated lemons. In 2020, the trial consisted of 2 replicates for both untreated and treated lemons. For untreated lemons, each replicate consisted of 10 whole fruit and 10 peel segments per sun-exposure time period, while for the treated lemons, each replicate consisted of 3 whole fruit and 3 peel segments. A trial using oranges was conducted in 2019 and consisted of 5 replicates for both untreated and treated fruit. For each replicate of untreated and treated oranges, 10 whole fruit and 10 peel segments were examined per exposure time period.

### 4.3. Sunlight Exposure of Citrus Pulp

**Figure 2:**
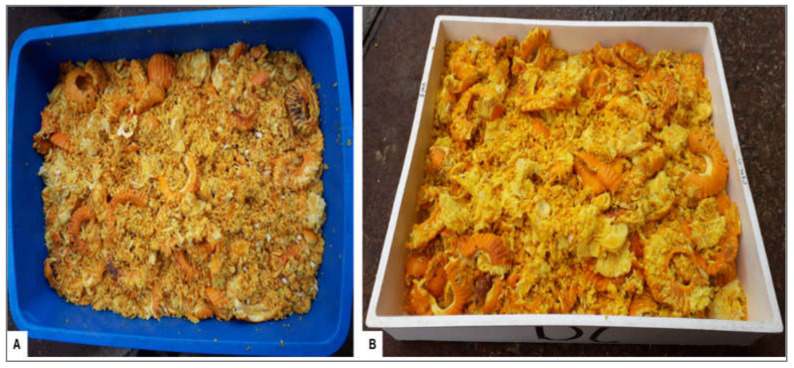
Citrus pulp generated from processing ‘Midknight’ Valencia oranges. (**A**) Pulp in a container and left overnight after fruit processing. (**B**) Pulp spread flat in an open tray and exposed to natural conditions.

Batches of treated and untreated (lemons and oranges) fruit were processed for juice extraction (JBT Citrus Juice Extractor, John Bean Technologies (Pty) Ltd., Cape Town, South Africa). The citrus pulp was in the form of peel segments, rag, and seeds (Figure 2). After fruit processing, the pulp was left overnight in containers (Figure 2A) and was then spread flat (layer of ±4 cm thick) in open trays (measuring 52 cm × 41 cm × 8 cm) the next day (Figure 2B). The pulp in different trays was exposed to sunlight either for 1, 2 or 3 days before 100 intact CBS lesions were counted from the peel segments that were exposed to sunlight (i.e., on the top of the layer) and a score was made regarding whether pycnidia were present or absent on each lesion. Lesions were also counted from pulp not exposed to sunlight (0 days). Twenty lesions with pycnidia, from each tray, were then investigated for pycnidiospore release and pycnidiospore germination. Seven trays with pulp were used for each sun-exposure period for both treated and untreated lemons and oranges. Trials were conducted once on lemons and repeated on oranges.

### 4.4. Pycnidiospore Release and Viability of CBS Lesions

To stimulate pycnidiospore release from pycnidia contained in lesions, a solution made of Valencia juice and citric acid was used. This solution was prepared by adding 50 mL of a 5% citric acid solution to 100 mL of freshly squeezed Valencia orange juice [24,27]. This solution was filtered through a 0.45 μm cellulose acetate membrane syringe filter (GVS filter technology) and stored in 2 mL Eppendorf tubes at 4 °C for not more than 24 h before use. A 20 μL drop of the Valencia juice and citric acid solution was placed on each selected lesion with pycnidia and was left for 10 min to allow spores to be released into the droplet. The droplet was drawn up using a pipette and spread onto water agar plates (WA, Difco^TM^, Becton, Dickinson and Company, Sparks, MD, USA). The water agar plates were examined using a stereo microscope (Zeiss Stemi DV4, Carl Zeiss (Pty) Ltd., Oberkochen, Germany) after 48 h of incubation at ±25 °C for the presence of *P. citricarpa* pycnidiospores. Due to the large number of plates that had to be assessed, germination was recorded but not quantified. If 1 germinating spore was observed on WA, the particular lesion was rated as viable. Single germinating spores were plated onto oatmeal agar plates (OA, Difco^TM^, Becton, Dickinson and Company, Sparks, MD, USA) to assess for *Phyllosticta* mycelium growth [37]. Images of pycnidiospores and those germinating on water agar were captured using a Zeiss Axiocam ERc 5s on a Zeiss Primo Star HAL light microscope (Carl Zeiss (Pty) Ltd., Oberkochen, Germany) at 20× magnification.

### 4.5. Verification of CBS Lesion Diagnosis

To confirm that lesions visually diagnosed as CBS lesions were caused by *P. citricarpa*, genomic DNA was extracted from 1 CBS lesion from a subset of fruit/peel segments from each treatment using the PROMEGA Wizard^®^ Genomic DNA Purification Kit (Promega, Madison, WI, USA) according to the manufacturer’s instructions. Real-time PCR to detect *P. citricarpa* was done using the KAPA PROBE FAST qPCR Master Mix, as well as primers and probes described by Hu et al. (2014) [38]. A plant cytochrome oxidase (COX)-based primer–probe set was used as a positive internal control to assess the quality of the extracted DNA [39]. The real-time PCR amplifications were performed using the Eco^TM^ Real-Time PCR System (Illumina, San Diego, California, CA 92122, USA) in a 20 µL reaction volume containing 2 μL of gDNA, 10 μL of 2× KAPA PROBE FAST qPCR Master Mix, 0.8 μL of each 10 μM primer (both COX and CBS primers), 0.4 μL of each 10 μM probe, and 6 μL of nuclease-free water. The standard amplification protocol was 95 °C for 3 min followed by 40 cycles at 95 °C for 15 s and 59 °C for 30 s. All reactions were performed in duplicate, and each run contained 2 negative controls (nuclease-free water served as a non-template control and gDNA extracted from CBS free fruit) as well as 1 positive control extracted from mycelium of *P. citricarpa*. The number of cycles required for the fluorescent signal to exceed the background signal, known as the quantification cycles, was used to indicate whether a sample was positive or negative for *P. citricarpa*. Fluorescence was measured in the green and yellow channels for CBS and COX, respectively.

### 4.6. Experimental Layout and Statistical Analyses

Trials were completely randomized. Data collected from treated and untreated fruit, peel segments, and citrus pulp consisted of the number of new CBS lesions per fruit and new lesions with pycnidia, percentages of new lesions with pycnidia per fruit, number of new lesions secreting pycnidiospores, and average number of germinating spores. The statistical analysis software Addinsoft XLSTAT Version 2020.4 (www.xlstat.com) was used to analyze data. Data and transformed data were typically variable and not normally distributed. The robustness of the F-test (ANOVA) was tested by Blanca et al. (2017) [40] under a wide variety of conditions involving non-normal distributions and was found to be a reliable and robust statistical procedure for use on data deviating from normality. Data were therefore subjected to ANOVA and significant differences between treatments were determined using Tukey’s HSD test, at a confidence interval of 95%.

## Figures and Tables

**Table 1:** Mean number of new CBS lesions and percentage of pycnidia-forming lesions per fruit on ‘Eureka’ lemons and ‘Midknight’ Valencia oranges after exposure to standard postharvest treatments consisting of pre-packhouse drench, chlorine wash, fungicide dips, and wax application, as well as a 5-week period of cold storage at 4 °C (oranges) and 7 °C (lemons) and 2 weeks at 25–27 °C under constant light and > 80% humidity.

Treatments	Mean Number of New CBS Lesions per Fruit		New Lesions with Pycnidia (%)
	2019	2020	
‘Eureka’ lemons *			
Untreated fruit	17.60 a	7.90 b	6.61 a
Treated fruit	2.10 c	1.30 c	1.40 b
‘Midknight’ Valencia oranges **			
Untreated fruit	14.21 a	–	4.43 a
Treated fruit	2.33 b	–	0.77 b

* Trials were conducted in 2019 and 2020 on lemons. Significant treatment × season interaction (*p* < 0.0001) was observed for number of new lesions, and a significant treatment effect (*p* < 0.0001) was observed for percentage of new lesions with pycnidia. ** Trial conducted in 2020 on oranges, and significant treatment effects (*p* < 0.0001) were observed for number of new lesions and percentage of new lesions with pycnidia. Means followed by the same letter for parameters are not significantly different per Tukey’s HSD test at a confidence interval of 95%.

**Table 2:** Mean percentage (standard error) of CBS lesions which released pycnidiospores on untreated ‘Eureka’ lemon and ‘Midknight’ Valencia orange fruit after exposure of lesions to sunlight from 0 to 6 h.

Sun-Exposure Period (Hours)	‘Eureka’ Lemon		‘Midknight’ Valencia Oranges	
	Untreated	Treated	Untreated	Treated
0	100.0 (0.000) a	0.0 (0.000) c	84.0 (3.685) a	0.0 (0.000) c
2	82.8 (5.003) b	0.0 (0.000) c	82.0 (3.861) a	0.0 (0.000) c
4	81.0 (5.193) b	0.0 (0.000) c	59.0 (4.943) b	0.0 (0.000) c
6	74.1 (5.800) b	0.0 (0.000) c	58.0 (4.960) b	0.0 (0.000) c

Means for each fruit type followed by the same letter are not significantly different (*p* < 0.05). Treated fruit were exposed to standard packhouse treatments (pre-packhouse drench, chlorine wash, fungicide dips, and wax application) before being subjected to 5 weeks cold storage. Untreated fruit were subjected to 5 weeks of cold storage without packhouse treatments.

**Table 3:** Mean percentage (standard error) of CBS lesions which released pycnidiospores on untreated ‘Eureka’ lemon and ‘Midknight’ Valencia orange peel pieces in pulp from processed fruit after exposure of pulp to sunlight from 0 to 3 days.

	‘Eureka’ Lemon		‘Midknight’ Valencia Oranges	
Sun-Exposure Period (Days)	Untreated	Treated	Untreated	Treated
0	54.3 (4.225) a	4.3 (1.718) c	19.3 (3.346) a	8.6 (2.374) b
1	21.4 (3.480) b	3.6 (1.574) c	17.9 (3.249) a	7.9 (2.282) b
2	8.6 (2.374) c	1.4 (1.007) c	2.9 (1.413) b	1.4 (1.007) b
3	3.6 (1.574) c	0.0 (0.000) c	0.0 (0.000) b	0.0 (0.000) b

Means for each fruit type followed by the same letter are not significantly different (*p* < 0.05). Treated fruit were exposed to standard packhouse treatments (pre-packhouse drench, chlorine wash, fungicide dips, and wax application) before being subjected to 5 weeks of cold storage. Untreated fruit were subjected to 5 weeks of cold storage without packhouse treatments.
